# Colorimetry of Luminescent Lanthanide Complexes

**DOI:** 10.3390/molecules25174022

**Published:** 2020-09-03

**Authors:** Julien Andres, Anne-Sophie Chauvin

**Affiliations:** 1 Section of Chemistry and Chemical Engineering, École Polytechnique Fédérale de Lausanne, 1015 Lausanne, Switzerland; 2 Institut des Sciences et Ingénierie Chimique, École Polytechnique Fédérale de Lausanne, 1015 Lausanne, Switzerland

**Keywords:** lanthanide, europium, terbium, samarium, dysprosium, luminescence, colour, colorimetry

## Abstract

Europium, terbium, dysprosium, and samarium are the main trivalent lanthanide ions emitting in the visible spectrum. In this work, the potential of these ions for colorimetric applications and colour reproduction was studied. The conversion of spectral data to colour coordinates was undertaken for three sets of Ln complexes composed of different ligands. We showed that Eu is the most sensitive of the visible Ln ions, regarding ligand-induced colour shifts, due to its hypersensitive transition. Further investigation on the spectral bandwidth of the emission detector, on the wavelengths’ accuracy, on the instrumental correction function, and on the use of incorrect intensity units confirm that the instrumental correction function is the most important spectrophotometric parameter to take into account in order to produce accurate colour values. Finally, we established and discussed the entire colour range (gamut) that can be generated by combining a red-emitting Eu complex with a green-emitting Tb complex and a blue fluorescent compound. The importance of choosing a proper white point is demonstrated. The potential of using different sets of complexes with different spectral fingerprints in order to obtain metameric colours suitable for anti-counterfeiting is also highlighted. This work answers many questions that could arise during a colorimetric analysis of luminescent probes.

## 1. Introduction

Luminescent trivalent lanthanide (Ln) ions are well known for their unique spiked emission bands due to forbidden *f-f* transitions [1]. Once coupled to organic ligands bearing a light-harvesting chromophore with suitable photophysical properties, most of the Ln ions are capable of producing luminescence by photosensitisation. This process is optimal for Ln(III) emitting in the visible spectrum, such as Eu, Tb, Dy, and Sm. Visible luminescent trivalent Ln ions are indeed easier to sensitise as chromophores absorbing in the UV from 250–380 nm are good energy donors for these Ln acceptors. Some chromophores are also able to sensitise near-infrared (NIR) emitters, e.g., Yb, Nd, and/or Er [2]. Nevertheless NIR-emitting Ln suffer from low intrinsic quantum yields and the measurement of NIR signals requires specific photodetectors sensitive to wavelengths > 800 nm. Eu and Tb are the most efficient emitters of the Ln series, with characteristic emission bands yielding red and green emission colours, respectively. Dy and Sm do not have that same emission intensity but can still yield colourful emissions perceivable by the human visual system (yellow and orange, respectively). Dy can, for example, yield white emissions when mixed with the blue emission of some ligands or of the matrix [3].

Ln ions are often cited to have excellent photophysical properties that are very attractive for imaging, colour reproduction, and lighting due to their distinctive colour purity [4,5,6]. The presence of Ln-doped materials in phosphors for cathode ray tubes (CRTs), plasma displays, and so-called fluorescent light bulbs certainly comforts this claim. Ln-based luminescent materials are also particularly suited for producing trichromic light emissions (red, green, blue aka RGB systems) by mixing different Ln ions, mostly due to the purity of the Eu(III), Tb(III), and Eu(II) red, green, and blue characteristic emissions [7,8,9,10]. Most Ln-based phosphors are composed of inorganic materials, but organic complexes or metal–organic frameworks (MOFs) show growing interests for organic light-emitting diodes (OLEDs) due to the larger derivatisation ability and variations of chemical structures [10,11,12,13,14].

Ln ions are usually found as trivalent Ln(III) ions, which is the most stable oxidation state across the Ln series of elements. However, some Ln ions have accessible divalent or tetravalent oxidation states that can be stable under certain conditions. A famous example is Eu(II), which is found in the blue luminescent BaMgAl_10_O_17_:Eu phosphor. New exciting developments have also been achieved in the field of divalent Eu(II) chemistry [15,16,17]. Furthermore, it was shown that photoinduced electron transfer from the excited photosensitiser to some Ln(III) ions, thereby forming divalent states, probably plays a role in the photophysical properties of a lot of luminescent Ln complexes [18,19].

The advantages of Ln luminescence over other luminophores are numerous. The well-separated spectroscopic levels of the lanthanide ions in addition to high antenna-generated shifts (sometimes referred to as Stokes shifts) makes them more resistant to quenching and photoredox processes, especially when complexed by protecting ligands that fill their high coordination number (typically around 9). Their emission peaks are few affected by the ligands due to the internal character of the involved *f* orbitals. The symmetry of the complex splits the spectroscopic levels differently and modulates the intensity of some transitions, but their energy stays very similar (usually never further away than 1 nm from the expected wavelengths) [1]. As a consequence, their colour is believed to be quite constant and hence to foremost depend on the nature of the Ln ion. The colour shift that can be obtained by variation of the ligand field is not well established. A theoretical study looked at the CIE coordinates of lanthanide-doped materials from the Judd–Ofelt parameters and obtained a good match between predicted and measured colours [20]. This study pointed out that other phenomena, such as line widening and Stark splitting, were neglected. In general, it is not clear to what extent instrumental parameters, such as slit openings (spectral bandwidth), or miscalibration errors, such as the wavelength accuracy and instrumental correction function, may affect the colorimetric analysis of Ln ions.

Complexes based on dipicolinate derivatives are efficient and versatile ligands for complexing and sensitising lanthanide ions [21,22,23,24,25,26,27,28,29,30,31,32]. In a previous work, we showed that they can be printed on paper and combined to obtain invisible full colour images for anti-counterfeiting of valuable documents [9]. In the present work, we extended the investigation of the colours of luminescent trivalent lanthanide complexes by looking at the spectral fingerprints of Eu, Tb, Dy, and Sm in three different types of complexes dissolved in aqueous solution. The influence of the Ln environment on the resulting colours will be illustrated. The effect of spectrophotometric parameters, such as the spectral bandwidths of the spectrophotometers, the sensitivity correction function, and wavelength accuracy, will be tested. The effect of the intensity units will also be analysed by omitting the conversion of spectral data to relative radiometric units. The natural output mode of modern spectrophotometers is indeed counts per seconds, which is a unit proportional to photon counts, whereas colour theory is based on radiometric spectral data. Finally, we will show the entire colour ranges (gamuts) of the different combinations of Eu and Tb luminescent red and green primaries (made of the different types of complexes) with a fluorescent blue primary. Considerations on the generation of white light, and how the choice of a reference white stimulus affect the gamuts will be discussed.

### Colour Theory

Colour science studies how light stimuli are perceived by the human visual system [33]. Colours are scientifically characterised as coordinates in a specific colour space. Several colour spaces are regularly used in colour science: CIE-RBG, CIE-XYZ, CIELAB, and CIELUV are typical colour spaces defined by the International Commission on Illumination (CIE, from French “Commission Internationale de l’Eclairage”) and using different sets of colour coordinates (red, green and blue *R, G, B*; tristimulus values *X, Y, Z*; lightness L*, green-red and blue-yellow axes a*, b* or u*, v*). Among them, CIE-XYZ plays a central role. It is a device-independent colour space and therefore acts as a connection space between device-dependent colour spaces. The tristimulus values *X*, *Y* and *Z* of the CIE-XYZ colour space are generally obtained from spectral data, *S*(*λ*), by applying colour matching functions ($\bar{x}(\lambda ),\bar{y}(\lambda ),\bar{z}(\lambda )$). A normalisation of the tristimulus values by their sum value gives the familiar CIE-xy chromaticity diagram. This chromaticity diagram does not take into account brightness, luminance, or lightness and therefore only represents colours in a flat 2-D space. A more uniform version of the chromaticity diagram called the “uniform chromaticity scale” (or CIE 1976 UCS) and defined by *u*′ and *v*′ values instead of *x* and *y* was developed and should be preferred for representing colours in a 2-D space because of its better uniformity [34].

For colour reproduction purposes, when colour mapping from an input device to an output device is required, CIELAB and CIELUV are the go-to spaces. They are both device-independent colour spaces that are more uniform than CIE-XYZ relative to the colour difference and both perform a different white point adaptation. CIELAB and CIELUV are defined relative to CIE-XYZ by a series of conditional transformations. The entire colour range that can be reproduced by a specific device is called a colour gamut. Different devices commonly have different gamuts. To standardise the colour gamut of display devices, standard gamuts, such as sRGB (standard Red Green Blue) were defined [35]. The sRGB gamut represents only part of the colours that can be perceived by the human visual system but covers most of the reproduction capability of most display devices. Nowadays, the standard for display devices tends to be shifted towards wider gamuts, such as AdobeRGB, DCI-P3, or Rec. 2020 [36]. In this work, full 3-D colour gamuts will be represented in the CIELAB colour space.

A lot of colour spaces define the values of their white point based on a standard reference illuminant. There are several classes of standard white reference illuminants (A, B, C, D, E, F, and LED). The most used illuminants are the “E”, “A”, and “D65” illuminants. Illuminant E consists in a continuous flat energy stimulus and has (*x*;*y*) coordinates of (1/3;1/3). Illuminant A simulates a tungsten-filament light bulb by a black body radiation at a temperature of 2856 K (*x*;*y*) = (0.44757;0.40745). The “D” illuminants simulate daylight. There is a series of D illuminants with several correlated colour temperatures. D65 is the most widely used reference white D illuminant with a correlated colour temperature of 6504 K and (*x*;*y*) = (0.31271;0.32902). D65 is also the reference white point of the sRGB colour space. The F series of illuminants are more specialised since they represent fluorescent lighting, similarly to LED illuminants. In this study, illuminants E and D65 will be used.

## 2. Materials and Methods

### 2.1. Preparation of the Ln Complexes

[Ln_2_(L^c2^)_3_] and [Ln(do3a-c)] were prepared and measured elsewhere [30,37,38].

The Na_3_[Ln(dipic)_3_] complexes were freshly prepared from 99.9% LnCl_3_ • 6 H_2_O salts, dipicolinic acid, and sodium carbonate (commercial product used as such) from Sigma-Aldrich (Buchs, St. Gallen, Switzerland), as described elsewhere [9]. The recrystallized complexes were then used to prepare 5 mM aqueous stock solutions of [Ln(dipic)_3_]^3−^. Diluted 0.1 mM solutions in Tris 0.1 M, pH 7.4 were finally prepared from these stock solutions.

### 2.2. Measurement of the Emission Spectra

[Ln_2_(L^c2^)_3_] and [Ln(do3a-c)] were prepared and measured elsewhere [30,37,38].

The emission spectra of [Ln(dipic)_3_]^3−^ were recorded at room temperature on a Horiba-Jobin Yvon Fluorolog FL-3-22 fluorimeter using 1-cm quartz Suprasil^®^ cuvettes and the diluted 0.1 mM solutions in Tris 0.1 M, pH 7.4. The spectra were corrected by the instrumental correction function.

The measured data points of the emission spectra are available in the Supplementary Materials.

### 2.3. Data Treatment

#### Extraction of the Ln Fingerprints and Conversion to Relative Spectral Irradiances

The Ln spectral fingerprint was extracted in Origin by fitting the spectrum of a non-luminescent analogue complex, such as Lu or Gd, on the ligand emission of the luminescent complex and then subtracting it. The baseline was further adjusted to zero by subtracting the average baseline value of the fingerprints. The spectral fingerprints were then imported in Matlab where the rest of the data treatment was performed.

Relative spectral irradiances were obtained from the imported Ln spectral fingerprints by multiplying them by 1/*λ* and then renormalizing.

### 2.4. Broadening Simulation

The broadenings of the spectral bandwidth of the Ln spectra were simulated by applying a Gaussian function with *σ* = 10 nm at each recorded wavelength and integrating the signal over the adjacent wavelengths (Matlab function normpdf(x,mu,sigma), where x is the wavelength matrix of the spectra, mu is the central wavelength on which the broadening is applied, and sigma is the standard deviation, i.e., bandwidth, of the Gaussian function).

### 2.5. Wavelength Shifts

Wavelength shifts were simulated by shifting the whole spectrum to ±2.5 nm.

### 2.6. Colorimetric Analysis

The colorimetric analysis was performed by cropping the spectra from 390–780 nm (setting out-of-range values to 0) and then applying the CIE-XYZ colour matching functions ($\bar{x}(\lambda ),\bar{y}(\lambda ),\bar{z}(\lambda )$) according to Equation (1). The *S_refW_*(*λ*) spectrum was chosen as a flat energy E illuminant reference. From the resulting tristimulus values, (*x*; *y*) and (*u*′; *v*′) chromaticity values were calculated from Equations (2) and (3):

(1) $$\begin{array}{l} X=k\cdot \int_{390}^{780}\bar{x}(\lambda )\cdot S(\lambda )\cdot d\lambda \\ Y=k\cdot \int_{390}^{780}\bar{y}(\lambda )\cdot S(\lambda )\cdot d\lambda \\ Z=k\cdot \int_{390}^{780}\bar{z}(\lambda )\cdot S(\lambda )\cdot d\lambda \\ k=\frac{100}{\int_{390}^{780}\bar{y}(\lambda )\cdot S_{refW}(\lambda )\cdot d\lambda } \end{array}$$

(2) $$\begin{array}{l} x=\frac{X}{X+Y+Z}\\ y=\frac{Y}{X+Y+Z}\\ z=\frac{Z}{X+Y+Z}\\ x+y+z=1 \end{array}$$

(3) $$\begin{array}{l} u^{\prime }=\frac{4x}{-2x+12y+3}\\ v^{\prime }=\frac{9y}{-2x+12y+3} \end{array}$$

The normalised Eu and Tb fingerprints were set as red and green primaries and combined with the normalised blue fluorescent emission of [Lu_2_(L^c2^)_3_], set as the blue primary. The linear combination was done by iterative scaling of each primary by increments of 5%, thus generating 9261 different “virtual” spectra. These spectra were converted to CIE-XYZ tristimulus values and then to CIELAB colours according to Equation (4). Different reference white points (*X_w_*;*Y_w_*;*Z_w_*) were tested for the conversion to CIELAB colours. In addition to the normalised primaries, the combination producing the same CIE-xy chromaticity as a D65 illuminant and an E illuminant were chosen. These two combinations were found by minimising the sum of the squares of the difference between the xyz chroma of the scaled white stimulus and the reference chromaticity of the desired illuminant using Matlab’s fmincon function. Full 3-D gamuts were plotted in Matlab using a “Ball-Pivoting algorithm” on the 9261 CIELAB points previously generated [39]:

(4) $$\begin{array}{l} L^{*}=116\cdot f\left(\frac{Y}{Y_{w}}\right)-16\\ a^{*}=500\cdot \left(f\left(\frac{X}{X_{w}}\right)-f\left(\frac{Y}{Y_{w}}\right)\right)\\ b^{*}=200\cdot \left(f\left(\frac{Y}{Y_{w}}\right)-f\left(\frac{Z}{Z_{w}}\right)\right) \end{array}\;\;\;\;f\left(t\right)=\left\{ \begin{array}{ll} \sqrt[{3}]{t} & \mathrm{if}\;t>{\left(\frac{6}{29}\right)}^{3}\\ \frac{t}{3\cdot {\left(\frac{6}{29}\right)}^{2}}+\frac{4}{29} & \mathrm{otherwise} \end{array}\right..$$

The CIELAB points can be converted to a cylindrical representation called CIELCh, where the radius represents chroma C*, and the angle hue *h*^0^. The CIELCh representation was used to represent slices of constant hues of full 3-D colour gamuts:

(5) $$\begin{array}{l} C^{*}=\sqrt{{\left(a^{*}\right)}^{2}+{\left(b^{*}\right)}^{2}}\\ h^{0}=\mathrm{atan}2\left(b^{*},a^{*}\right) \end{array}$$

## 3. Results and Discussion

### 3.1. Colours of the Visible Ln(III) Ions and Variations Induced by the Ligand Field

The aim of this part is to compile the colours that can be obtained by using different Ln ions in different ligand environments, thereby exploring the colorimetric shift that can be induced by variations of the ligand field.

**Scheme 1:**
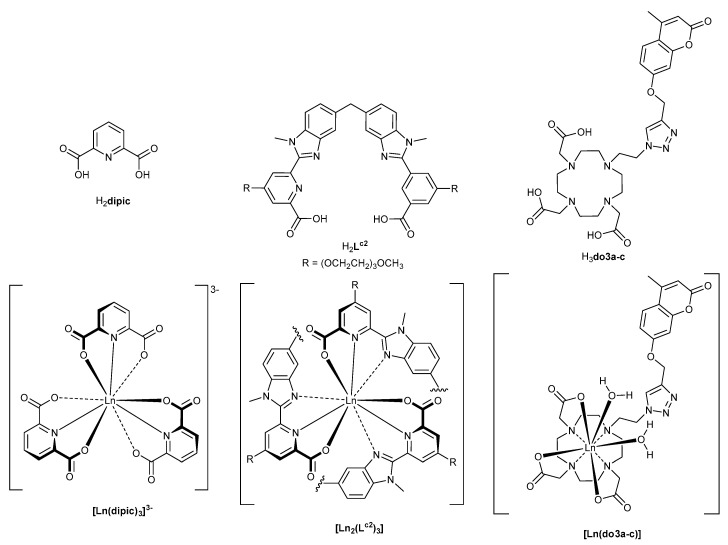
Structure of dipicolinic acid (H_2_**dipic**) of the helicate ligand (H_2_**L^c2^**) and of the do3a ligand (H_3_**do3a-c**) forming 1:3, 2:3, and 1:1 (Ln/L) complexes with a D_3h_, D_3_, and C_4v_ symmetry around the Ln ion, respectively (Δ enantiomer shown).

The emission spectrum of three series of water-soluble Ln(III) complexes were analysed. Each series includes the Eu(III), Tb(III), Sm(III), and Dy(III) complexes of a given ligand. The first series corresponds to trisdipicolinate lanthanide complexes [Ln(dipic)_3_]^3−^ with a D_3h_ symmetry around the Ln ion. The second series includes the neutral dinuclear helicate complexes [Ln_2_(L^c2^)_3_], which have a D_3_ symmetry around the Ln ions. The third series is made of a 1,4,7,10-tetraazacyclododecane-1,4,7-triacetic acid (do3a) derivative bearing a coumarin-appended side chain (do3a-c ligand). The do3a-c ligand forms a dihydrated neutral 1:1 complex [Ln(do3a-c)(H_2_O)_2_] with a C_4v_ symmetry around the Ln ion. The structures of the complexes are shown in Scheme 1.

These complexes, being in solutions, may be in equilibrium between several conformations and species. The measured luminescence is hence an average signal representative of this equilibrium [40,41]. There is also significant residual fluorescence in some of the complexes, i.e., [Dy_2_(L^c2^)_3_], [Sm_2_(L^c2^)_3_], and all the [Ln(do3a-c)(H_2_O)_2_], indicative of an incomplete photosensitisation of the Ln ions by the fluorescent chromophore. In these complexes, the Ln fingerprints were first extracted by normalizing and subtracting the fluorescence emission. This ensured that the colorimetric analysis is independent of variations in fluorescence (both in terms of shape and intensity) and thus is representative of the Ln ion in analogous ligand environments. The resulting extracted Ln spectra are similar in shape to time-resolved spectra that would be recorded after pulsed excitation.

**Figure 1:**
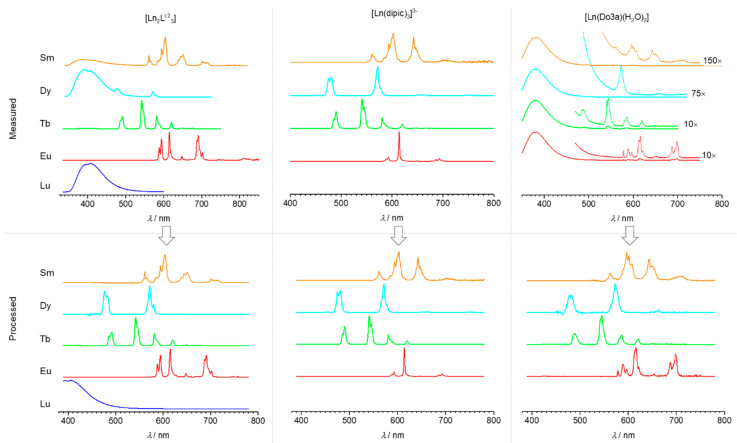
Spectral data in normalised counts per second (measured) and final cropped relative spectral irradiances (processed) for the 3 series of complexes.

The Ln spectra were converted to radiometric units (relative spectral irradiance). The intensity in counts per second (corrected to account for the wavelength sensitivity of the instrument) was multiplied by 1/*λ* (energy of a photon is equal to *hν* = *hc*/*λ*, where *h* is Planck’s constant, thus proportional to the frequency *ν* or inversely proportional to the wavelength *λ*) and then normalized so that the maximum intensity is unity. The same procedure was applied to the blue fluorescent emission of the non-emissive Lu complex [Lu_2_(L^c2^)_3_]. This complex served as a reference of typical fluorescence and as a blue emitter, which is not achievable with trivalent Ln luminescence. The blue fluorescence of this Lu complex comes from the chromophore on the ligand and not from the Ln ion. Gadolinium or lanthanum complexes are alternatives that produce comparable results as these Ln centres are non-luminescent, at least under the usual low-energy UV conditions studied here. The normalised counts per second spectra (measured) and the resulting (processed) relative spectral irradiances of the complexes are displayed in Figure 1.

The characteristic transitions of Eu, Tb, Dy, and Sm are well recognisable in the three series of complexes. Eu has four to five visible transitions, with the hypersensitive ^5^D_0_→^7^F_2_ transition centred around 615 nm being the most intense one and the ^5^D_0_→^7^F_1_ and ^5^D_0_→^7^F_4_ transitions also clearly visible. Tb has four major visible transitions, with the ^5^D_4_→^7^F_5_ transition centred around 545 nm being the most intense of the lot. Dy has two main bands, one centred at 477 nm (^4^F_9/2_→^6^H_15/2_ transition) and the largest one at 572 nm (^4^F_9/2_→^6^H_13/2_ transition). Sm has four visible bands, with the one centred at 603 nm (^4^G_5/2_→^6^H_7/2_ transition) being the most intense of the four.

The colorimetric analysis was then performed by multiplying the relative spectral irradiance *S*(*λ*) by the CIE-XYZ colour matching functions $\bar{x}(\lambda ),\bar{y}(\lambda ),\bar{z}(\lambda )$, integrating the resulting spectra between 390 and 780 nm and multiplying by a factor *k* defined as 100 divided by the *Y* response of a reference white spectrum *S_refW_*(*λ*) (here an E illuminant, i.e., with a unity flat relative to spectral irradiance), as defined in Equation (1).

**Figure 2:**
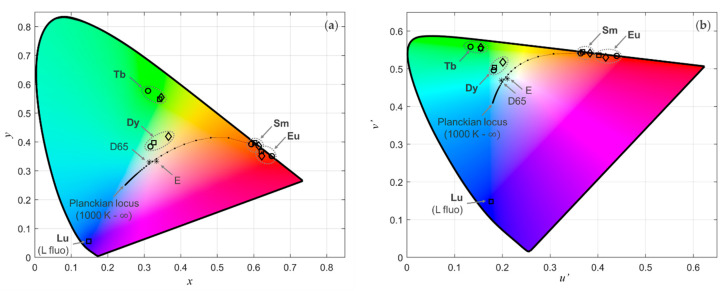
Chromaticity diagrams CIE-xy (**a**) and CIE 1976 UCS (**b**) of the 3 series of Ln complexes (circle = dipic, square = L^c2^, diamond = do3a) with the blue fluorescent colours of [Lu_2_(L^c2^)_3_], D65 and E illuminants and the Planckian locus showing the colours of black body radiations from 1000 K to ∞.

These CIE-XYZ tristimulus values were then converted to CIE-xy chroma values by dividing them by the sum of the *X*, *Y*, and *Z* coordinates. These (*x;y*) coordinates are typically represented on a chromaticity diagram showing the *x* and *y* values together with the locus of perceivable monochromatic colours and purple axis. The CIE-xy chromaticity diagram is a convenient figure to visualise individual colours but is not a good representation of colours when trying to compare them together. A simple transformation of the CIE-xyz values to *u*’ and *v*’ chroma values enable display of a more uniform representation of the different colours that can be perceived in a CIE 1976 uniform chromaticity scale (UCS) diagram. The colours of the Ln complexes of the three series are shown on both diagrams in Figure 2.

The different environment around the complexed Ln ion results in subtle differences in their emission spectra due to the ligand field (see Figure 1). The transition energies are little affected by the ligand field because of the internal character of the involved *f-f* transitions. On the other hand, the transition probabilities could be drastically different. This is especially the case when the Ln ion possesses hypersensitive transitions (i.e., with intensities that are very sensitive to the environment). The consequence of that is that colour changes are usually minor within a series of complexes for a given Ln. Here, this is clearly the case as all the characteristic spectral fingerprints in Figure 1 have superimposable bands in terms of wavelengths but with some fluctuations in their relative intensities. The emitted colour remains red-orange for europium, green for terbium, yellow to white for dysprosium, and orange for samarium (see Figure 2). The most visible difference of ligand-induced colour shifts is for Eu(III), as expected by its hypersensitive ^5^D_0_→^7^F_2_ transition, which can drastically change in intensity relative to the other bands. The narrow peak-like nature of this transition in the dipicolinate complex dominates compared to the other transition and gives the complex a very pure red emission. On the other hand, the complex of the helicate appears on the orange side, closer to the colours of the samarium complexes. Tb has a greener luminescence in the trisdipicolinate environment than in the do3a or bimetallic tris-L^c2^ complexes, which is a consequence of weaker ^5^D_4_→^7^F_4_ and ^5^D_4_→^7^F_3_ transitions together with a slightly higher ^5^D_4_→^7^F_6_ transition compared to the reference ^5^D_4_→^7^F_5_ transition. Dy is a bit yellower in do3a, which is due to a slightly weaker ^4^F_9/2_→^6^H_15/2_ transition relative to the reference ^4^F_9/2_→^6^H_13/2_ transition. Nevertheless, the resolution of the Dy spectrum is lower in the do3a complex, due to the much lower quantum yield of the Dy emission in this environment, i.e., 5× smaller than the corresponding [Eu(do3a-c)] and [Tb(do3a-c)] complexes [30]. This might introduce some uncertainty concerning the resulting colour of the Ln fingerprint.

Eu, Tb, and Sm can be considered as emitters with rather “pure” colours because they are located close to the edge of the UCS diagram, which represents colours obtained by using monochromatic stimuli. Dy, because of its “two-bands” nature with one in the blue and one in the yellow, emits light closer to white stimuli that can be yellow or blue shifted depending on the ratio of the two bands. Eu is the most sensitive Ln ion of this series regarding the influence of the ligand field because of its hypersensitive transition. Maximising the intensity of a single transition is desirable as it reinforces the colour purity of the emission. Overall, the dipicolinate environment with its D_3h_ symmetry seems to produce the largest colour gamut, as seen by the larger triangle delimited by the dipic Eu, Tb, and blue points in Figure 2. Finally, high quantum yields are desirable, not only because they produce more intense overall signals but because they guarantee that minimal residual fluorescence are present and that the Ln fingerprints are well resolved by the spectrophotometer.

### 3.2. Effect of the Bandwidth on the Calculated Ln Luminescent Colours

**Figure 3:**
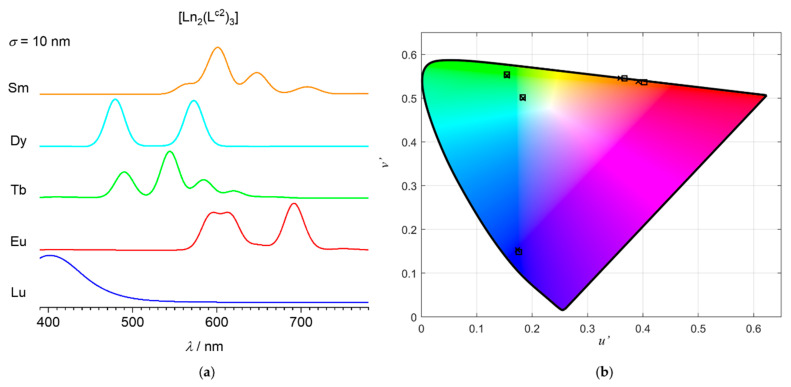
(**a**) Simulated relative spectral irradiances for broadened Ln spectra (Gaussian function with *σ* = 10 nm) and (**b**) corresponding colours (crosses) compared to the colours obtained from the original spectrum shown in Figure 1 (squares).

The spiked nature of Ln emissions makes them particularly susceptible to measurement broadening due to the bandwidth of the spectrometer. Low-quantum-yield Ln ions, such as Dy and Sm, often require opening of the slits of the emission monochromator in order to obtain good clean signals. This typically broadens the different bands of the luminescent signal and induces a loss of resolution and a shift of the local maxima. This effect can be simulated by taking a reference spectrum and applying a Gaussian bandpass filter centred at the different wavelengths of the spectrum. The effect of such broadening on the emission colour of the Ln ions could then be calculated and is displayed in Figure 3. These spectra are comparable with the original relative spectral irradiances in Figure 1.

The effect of a large 10-nm broadening is clearly visible on the emission spectra. The fine structure of each transition, as well as small transitions, are lost in broadbands. The peak intensity of these broadbands can also change. This is observed, for example, on the Eu spectra. As shown in Figure 3, the effect of the broadening of the spectral bandwidth does not correlate with a significant difference on the CIE UCS diagram. This result is important as it means that colorimetric tests done with Ln ions should not be very sensitive to the bandwidth setup of the photodetector.

### 3.3. Effect of the Spectrometer Correction Function and Spectral Radiometric Conversion on the Calculated Ln Luminescent Colours

Producing reliable emission spectra that are independent of the spectrometer is only possible if the instrument is properly calibrated both in terms of the wavelength accuracy and wavelength sensitivity. Here, the effects of miscalibrated instruments on the colorimetric data of Ln ions were tested by not applying the instrument correction function, by shifting the wavelengths, and by not performing the conversion from relative photon counts to relative radiometric units. The data of the [Ln_2_(L^c2^)_3_] series were used.

**Figure 4:**
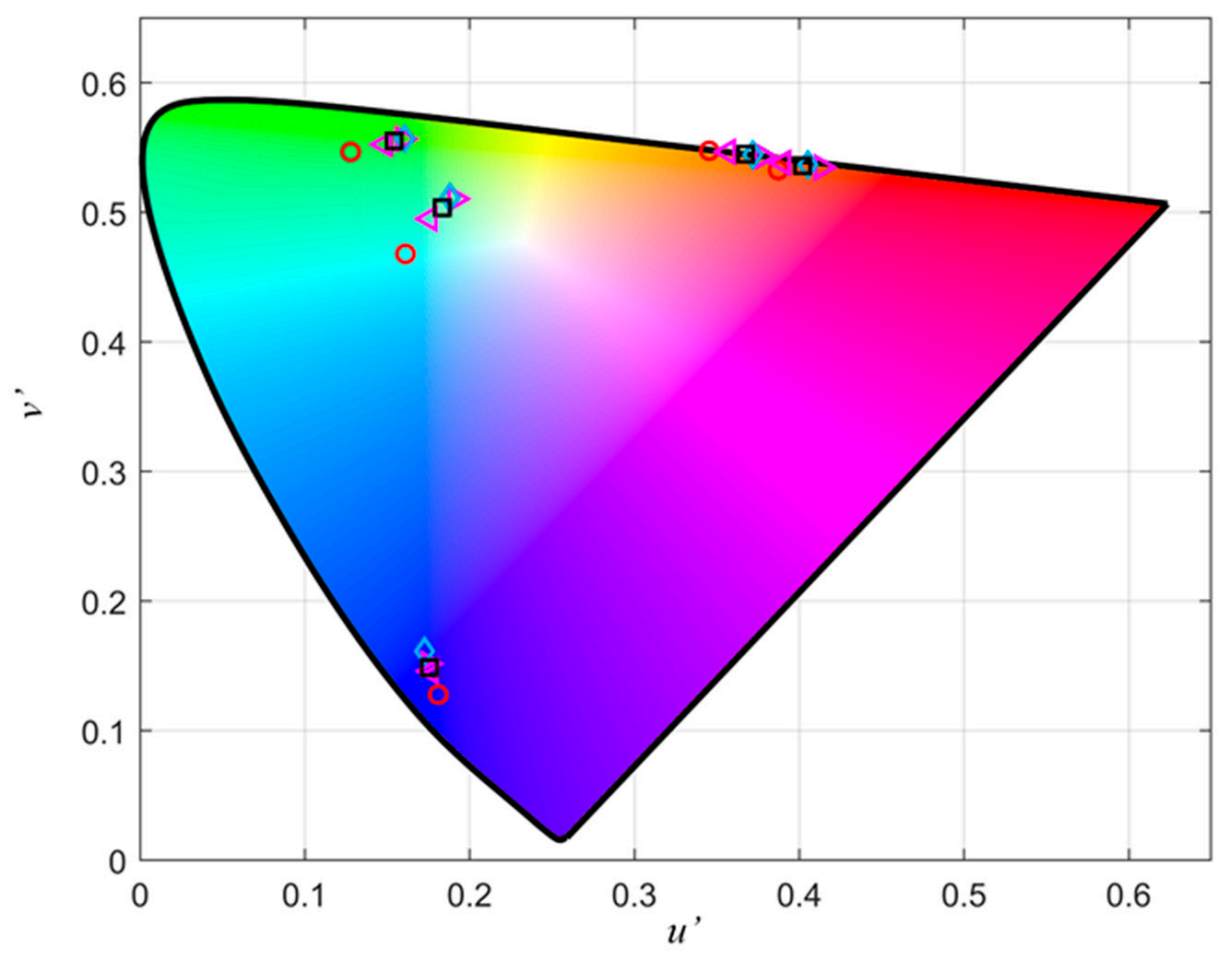
Miscalibration effects on the UCS of the [Ln_2_(L^c2^)_3_] series for unapplied instrumental correction function (red circles), wavelength shifts of ±2.5 nm (magenta triangles), and spectral data in normalised photon counts (blue diamonds) compared to the correct values (black squares).

The results are shown in Figure 4. The black squares represent the accurate colour points that were already presented in Figure 2, the red circles the colours that would be obtained without applying the instrumental correction function, the blue diamonds the colours from spectra in counts per seconds instead of relative spectral irradiances, and the magenta triangles the colours upon shifting the wavelength accuracy by ±2.5 nm. The red circles are consistently further away from the black squares than the other points. This indicates that the most important parameter for obtaining accurate colours is the application of the instrument correction function. Concerning the wavelength accuracy, the biggest wavelength shift that was tested here (magenta triangles) is ±2.5 nm. This shift is quite large but gives chroma differences that are comparable to the chroma difference induced by forgetting the radiometric conversion of the spectral data (blue diamonds). Smaller wavelength shifts would produce even smaller differences. Here, Ln ions have an advantage over broadband fluorescence from organic fluorophores as they can be used to calibrate the wavelengths of the spectrometers (wavelength calibration is routinely performed using holmium oxide glass or didymium glass, i.e., glass containing a mixture of Nd and Pr). This is possible because *f-f* transitions are always at the same energy.

The situation is similar with a typical blue fluorescence broadband, such as the one of the blue fluorescent emission of [Lu_2_(L^c2^)_3_]. The blue chroma is only slightly affected by shifts of the wavelength (±2.5 nm), slightly more affected by radiometric conversion, at least compared to the tested wavelength shifts, and finally significantly blue shifted if the correction function is not applied. This result can be rationalised because the correction function recovers some intensity in the green and red part of the spectra.

To sum up, instrument correction functions remain essential for obtaining accurate colorimetric data. Wavelength accuracy can be directly checked by using the energy of the Eu, Tb, Dy, or Sm *f-f* transitions and should be adjusted regularly in order to prevent the accumulation of errors. This is especially true for spectrometers that have monochromators as they are based on electrical motors that can drift over time. Finally, when calculating colour data, one needs to be sure that the colours are calculated from spectra in radiometric units. If a software or script is used to calculate colours from spectra, it means that one should know if the input spectra has to be relative counts per second data that are converted by the software or script, or if the input spectra has to be relative spectral irradiance, in which case the conversion must first be done manually.

### 3.4. Combinations and Gamut

#### 3.4.1. Combining Eu, Tb, Sm, and Dy for Colour Reproduction

Mixing complexes of different Ln ions can produce a whole range of luminescent colours. Nevertheless, if we only consider Eu and Tb complexes made of a single ligand, mixing these complexes would only produce shades of red, green, orange, and yellow, which all stand on a straight line in the UCS diagram between the two pure complexes (see Figure 2). As seen previously, we cannot rely on mixing ligands in order to produce large variations of luminescent colours on each individual Ln emission. Adding Sm only slightly increases the gamut, as the Sm chroma are very close to the “Eu-Tb line”. Dy can extend the gamut further, as it is clearly further away from this line, but the range of colours would be limited compared to a full gamut delimited by red, green, and blue primaries. Moreover, quantum yields can be drastically different, especially when comparing Sm and Dy with Eu and Tb complexes. Therefore, in order to obtain signals of a similar intensity for Dy, Sm, Eu, and Tb, the concentration of the complexes would have to be adjusted potentially to drastically different values. This would introduce issues due to the stability of the complexes and due to inner-filter effects. From a purely colorimetric and practical point of view, using Dy or Sm is thus not beneficial.

#### 3.4.2. Tuning of Red, Green, and Blue Primaries: Matching Intensities and White Points

The most efficient way to reproduce a wide range of luminescent colours is to use Eu as a red primary, Tb as a green primary, and a blue fluorescent compound as a blue primary. This RGB trichromic system enables reproduction of most of the colours that can be generated by a standard display device (sRGB). Here, we used the different Eu and Tb complexes (dipic, L^c2^, and do3a) as red and green primaries and the [Lu_2_(L^c2^)_3_] complex as a common blue-emitting primary. Other blue-emitting compounds with proper physicochemical properties could also be used though.

When combining luminescence, the relative intensity of the primary signals becomes important. The normalisation cannot be done individually on each primary but has to be done by the same value on all the relative intensities of the primaries. Therefore, the maximum intensities of the red, green, and blue primary luminescence have to be matched to a similar level prior to normalisation. Practically, this can be done by adjusting the concentration of the emitters in accordance with their quantum yields and extinction coefficients until the energy output matches.

**Figure 5:**
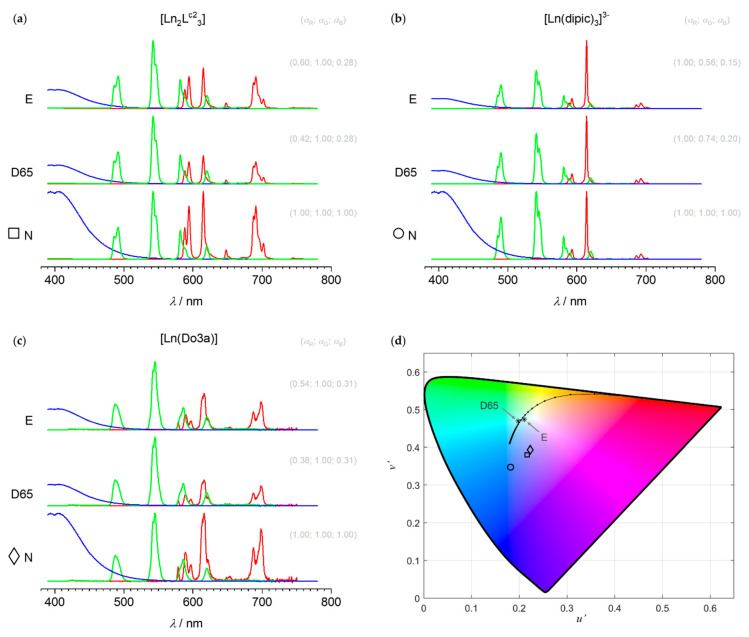
(**a**–**c**) Relative spectral irradiances of the tested R, G, and B primaries and (**d**) resulting (1; 1; 1) white points on the UCS insert for the N white points of the dipic (circle), L^c2^ (square), and do3a (diamond) series.

As a starting point, we chose the peak-normalised relative spectral irradiances of each primary {*S*_R,N_(*λ*); *S*_G,N_(*λ*); *S*_B,N_(*λ*)}as if they were previously matched in intensity. We then looked for other combinations of relative intensities that would give other white points. In order to describe the amount of each peak-normalised R, G, and B primary in the resulting spectrum, a vector (*a*_R_; *a*_G_; *a*_B_) was used. Each component of this vector is enclosed between 0 and 1. Two reference white points were targeted: An E illuminant and a D65 illuminant. We searched for (*a*_R_; *a*_G_; *a*_B_) combinations that would produce the same chromaticity as these reference white illuminants by minimising the sum of the squares of the difference between the xyz chroma of the scaled white stimulus and the reference chroma of the desired illuminant. Finally, these (*a*_R_; *a*_G_; *a*_B_) values were applied to the peak-normalised {*S*_R,N_(*λ*); *S*_G,N_(*λ*); *S*_B,N_(*λ*)} primaries in order to rescale the corresponding primaries and thus form new sets {*S*_R,E_(*λ*); *S*_G,E_(*λ*); *S*_B,E_(*λ*)} and {*S*_R,D65_(*λ*); *S*_G,D65_(*λ*); *S*_B,D65_(*λ*)} that would produce the targeted white point upon unity combination. The resulting primaries *S_i,w_*(*λ*) are shown in Figure 5.

Both the L^c2^ and do3a sets required a lowering of the intensities of the red and blue primaries in order to obtain either an E or a D65 white point. On the other hand, the dipic set kept the intensity of the red primary at its maximum value and decreased the intensities of the green and blue primaries. In all cases, the blue channel was greatly reduced (*a*_B_ between 0.15 and 0.31). This can be rationalised by the broad emission of the blue fluorescent primary compared to the narrow-spiked emissions of the Ln ions. Inversely, the very narrow nature of the [Eu(dipic)_3_]^3−^ emission means that it has to be maximal in order to have a proper contribution to the RGB mixing, thus imposing a maximisation of the red primary in the dipic set. This also explains why the peak-normalised set of primaries (N reference white) for the dipic complexes (circle in Figure 5) has such a blue-shifted colour compared to the chromaticity of the other peak-normalised sets (square and diamond).

#### 3.4.3. Combining Red, Green, and Blue: Full 3-D Colour Gamuts

The red, green, and blue primaries shown in Figure 5 were then linearly combined by increments of 0.05, resulting in 9261 spectra and thus luminescent colours. These spectra were converted to CIE-XYZ and then CIELAB using the (1; 1; 1) stimulus as the white reference point. The corresponding gamuts were constructed from the CIELAB points by using a “Ball-Pivoting algorithm” [39].

**Figure 6:**
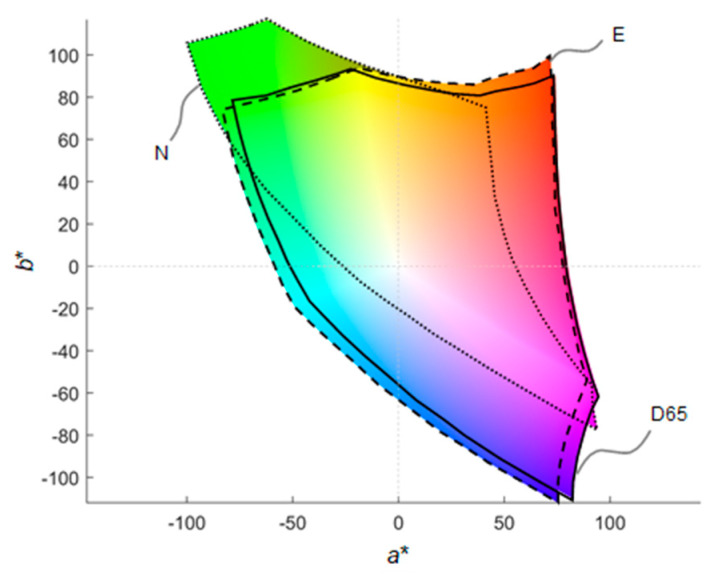
CIELAB projection of the full 3-D gamuts onto the a*b* plane for different white points (N, E, D65) of the dipic series.

In a first step, we looked at the colour gamuts produced by the dipic set of primaries upon variation of the reference white point (Figure 6). As seen on this figure, using the N reference white produces the narrowest gamut, while the E and D65 reference white points produce comparable gamuts. It can be observed in Figure 5 that *S*_R,N_(*λ*) + *S*_G,N_(*λ*) + *S*_B,N_(*λ*) is not a proper white point, despite being the most straightforward combination of R, G, and B spectra. This illustrates that a proper white point is needed in order to produce large gamuts obtained by the mixing of Eu and Tb spectra with a blue source.

**Figure 7:**
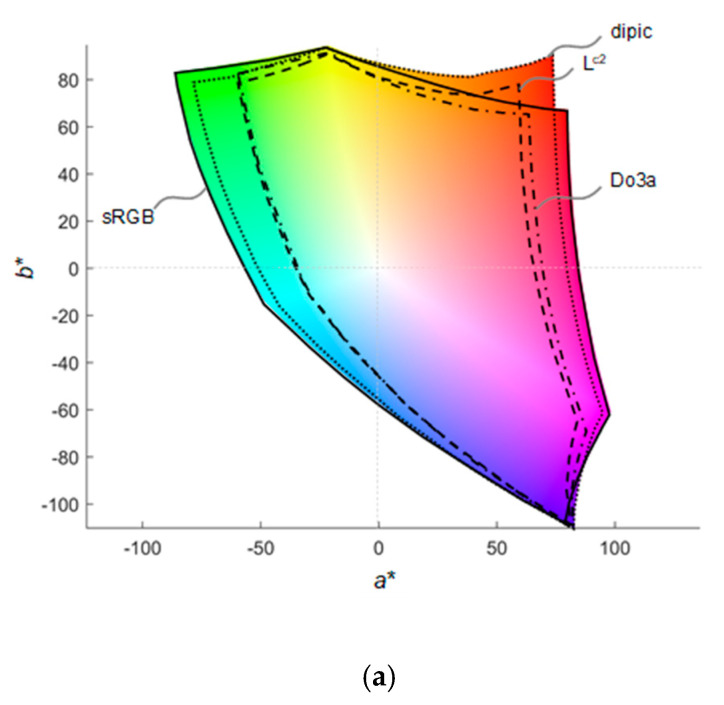

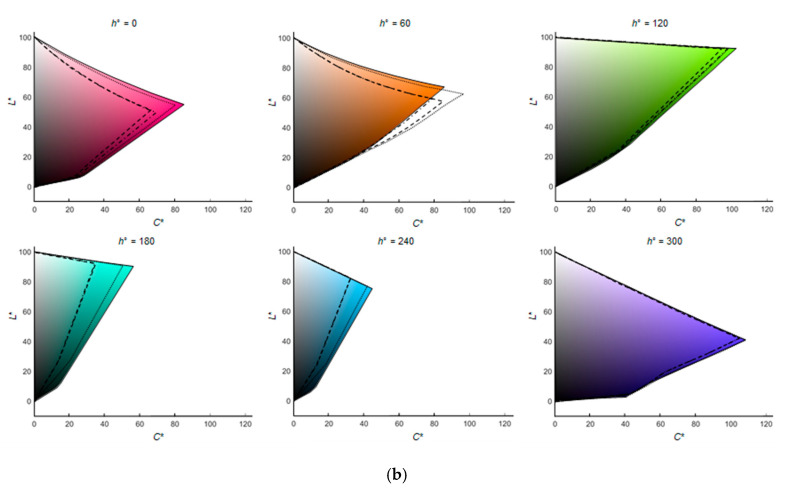
(**a**) CIELAB projection of the full 3-D gamuts (sRGB **—**, dipic **·····**, L^c2^ **– – –**, do3a **– · – ·**) onto the a*b* plane for the three series of Ln complexes with primaries combining to a D65 white point, and (**b**) corresponding hue cuts in CIELCh (cylindrical representation of CIELAB).

Figure 7 compares the influence of the ligand on the full 3-D colour gamut. We decided to fix the white point to D65, in order to have the same white reference as the sRGB gamut. This enabled us to compare the gamuts with a well-known reference. The dipicolinate series offers the widest gamut due to its remarkable red primary. This information is already visible on chromaticity diagrams (see Figure 2), as the triangle drawn from the three primary colours is larger in the case of the europium tris-dipicolinate complex. Compared to the sRGB gamut, which is a standard gamut for display devices, the dipic gamut is a little smaller in the green and cyan colours but larger than sRGB in the red and orange colours. The other two sets of ligands show gamuts that are, in general, smaller, especially along the a* axis. The L^c2^ ligand has a red primary, which is outside of sRGB but, as already discussed previously, shifted toward orange. The slightly greener primary of the dipic ligand was also already observed in Figure 2. As expected, the common blue primary means that the gamuts are similar in the blue colours (see hue, *h*° = 300 in Figure 7).

#### 3.4.4. Metamerism and Intensity Matching between Sets of Primaries

**Figure 8:**
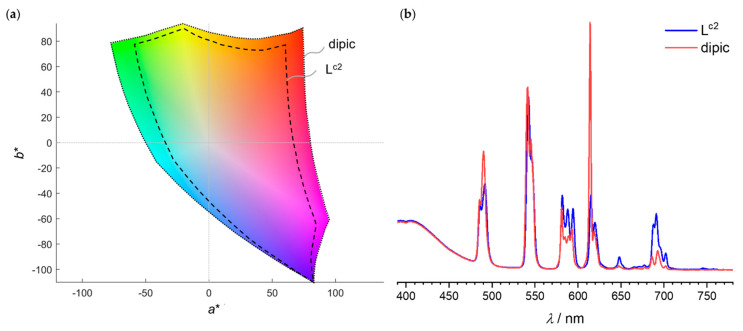
(**a**) Overlapping gamuts of the dipic and L^c2^ sets with the white points matched to have the same luminance *Y* and to have the same chromaticity as a D65 illuminant. (**b**) Corresponding white relative spectral irradiances where all the primaries of the L^c2^ set were scaled by a factor of 0.6989 in order to match the luminance of the dipic white spectrum.

A large overlap of the gamuts of the three sets of Ln complexes is observed in Figure 7. This is important because it means that the same colour can be reproduced by choosing either set of complexes, as long as this colour is located inside all three gamuts. This paves the way to anti-counterfeiting by metamerism (perception of the same colour from different spectral power distributions). The colours would indeed be perceived similarly by the human visual system, but the matched colours would have different emission spectra that could be identified by spectrophotometry or possibly by using filters (if sufficient contrast or colour shifts can be obtained this way). Because the different sets do probably not emit with the same brightness (quantum yield, extinction coefficient, and concentration may vary), matching of the intensity across the different sets would be required. This can be done by adjusting the concentrations and measuring the different sets (at least the three primaries and the white point) under the exact same conditions. Here, we simulated that by scaling the relative spectral irradiances of the D65-matched white points of the dipic and L^c2^ sets so that their luminance *Y* matched (Figure 8).

A seen in Figure 8, the resulting white stimuli are immediately recognisable when looking at their relative spectral irradiances due to the drastically different Eu fingerprint. The gamuts of the dipic and L^c2^ sets were recomputed to account for the new white points with identical luminance (Figure 8). It is now possible by minimisation to compute the colour combination of any CIELAB points enclosed within the smaller L^c2^ gamut and to find the corresponding dipic combination that would produce the same colour. For example, the red primary of the L^c2^ gamut corresponds to the CIELAB point (49.57; 59.88; 77.14). The same point in the dipic gamut can be reproduced by using the corresponding (R;G;B) combination (0.765; 0.031; 0.001), where R, G, and B are the components of a new set of primaries whose (1; 1; 1) combination produces a D65 white point with the same luminance as the (1; 1; 1) combination of the L^c2^ set.

#### 3.4.5. Discussion: From Computer Simulations to Real Applications

The model proposed here is of course an ideal situation where the emission is untainted by extra light coming either from the medium or from the excitation light source. This is expressed in the gamut as pure black points with 0 of luminosity. If the medium is slightly fluorescent upon excitation, or if some residual visible light coming either from the environment or from the excitation light source is measured, the blackest point is raised on the luminosity axis. Most spectrophotometers have good monochromatic excitation sources and do not allow any external light to contaminate the detector. Such issues could then arise within custom setups built on optical tables or with alternative light sources that do not use a monochromator. The most common excitation source in UV is the mercury lamp. A typical laboratory UV lamp can be switched from 254 to 365 nm and is usually filtered by a UV bandpass filter that removes most of the visible lines present in the emission spectrum of mercury. The 254-nm mode is faintly visible as blueish white light, whereas the 365-nm mode appears dark purple. These UV light source colours are due to residual emission in the visible spectrum. The effect of these residual emissions in a real-world setup is to slightly shift the primaries, therefore shifting the combination of primaries producing the desired white point, and secondly, to have a black spectrum that is not black anymore but often contains some of the residual light source emission. Real black points, being more or less luminous depending on the amount and nature of the residual undesired light and the amount of primary mixing required to compensate the colour of the black point, are thus often higher than zero on the greyscale axis. This phenomenon can be attenuated by limiting scattering through the medium and setup. When scattering within the medium or matrix is not controllable, e.g., when the medium contains particles or is a diffusive surface, such as paper, measurement with an aspecular angle between the light source and the detector should be preferred in order to eliminate the specular component of the reflected light. As demonstrated previously, good colour reproduction can still be obtained with non-zero black points due to the usage of a 254-nm mercury lamp [9].

Another important point in a real system would be inner-filter effects and non-linear mixing. This phenomenon appears when concentrations increase. The addition of one primary decreases the emission of a fixed primary due to the diminished amount of excitation light available. This behaviour can even be observed by increasing the amount of a single primary and observing the resulting emission. Eventually, the emission has to reach a plateau. This saturation usually indicates that the observed excited area absorbs all the available light. An alternative explanation is that quenching is involved. Quenching reduces the emission of one high-energy emission by transferring it to an available lower energy state of the quencher. The quencher is usually non-emissive and its addition results in a decrease of the emission of the donor state. Self-quenching can be produced if the emitter is able to deactivate its own emission in a concentration-dependent way. Quenching can be distinguished from inner-filter effects by lifetime measurements, as inner-filter effects do not produce any variation on lifetimes. In the case of photosensitised Ln complexes, the Ln emission is usually neither quenched nor self-quenched because the Ln emitter is protected from external energy acceptors by the ligand. On the other hand, the photosensitiser is more sensitive towards quenching and part of the energy can be lost before it has a chance to be transferred onto the Ln acceptor. Some Ln-based probes use this kind of interaction to be responsive to a desired biological, chemical, or physical event, interaction, reaction, or analyte [42]. Therefore, tests should be performed when mixing Ln complexes together or with other compounds in order to ensure that no significant quenching is happening. Quenching would indeed require an extensive modification of the colorimetric model to account for such a non-linear behaviour.

## 4. Conclusions

A colorimetric analysis of three series of Ln complexes was undertaken. The aim was to investigate the range of colours that can be obtained by using Ln ions emitting in the visible spectrum. Eu, Tb, Dy, and Sm were studied in three different environments: Within well-described trisdipicolinate complexes, within a typical do3a ligand, and within a supramolecular homobimetallic helicate architecture forming 2:3 Ln:L complexes. The ligand-centred blue fluorescence of the [Lu_2_(L^c2^)_3_] complex was also used as a reference blue emission.

After extraction of the Ln spectral fingerprints and conversion to normalised relative spectral irradiances, we showed that Ln fingerprints are not equal towards colour variations within different ligand environments. The colour shifts depend on the Ln ion and on which transitions are affected by the change of environments. Eu emission is orange-shifted within the L^c2^ and do3a structures compared to the dipic complex, which has a red emission closer to the monochromatic edge of the CIE 1976 UCS diagram. Tb is also greener in the dipic complex due to small intensity shifts of the “satellite” transitions around the major transition centred at 545 nm. This makes [Ln(dipic)_3_]^3−^ a better candidate for accurate colour reproduction compared to the other Eu complexes studied here. This effect was further demonstrated when looking at the CIELAB 3D gamuts of the three sets of ligands. The dipic gamut is larger than the other two sets and closer to the reference sRGB gamut. Dy and Sm showed similar colour shifts to Eu and Tb, with Sm displaying the most limited shifts of the series, probably due to the conjunction of limited variation of the intensity in the different transitions and limited sensitivity for variations in the red part of the spectrum.

The investigation of the spectral bandwidth and miscalibration of the spectrometer demonstrated that the spectral bandwidth does not drastically shift the colours of Ln ions or blue fluorescent signals, and that a proper instrumental correction function is the most important parameter to ensure that accurate colours are calculated from the spectral data.

Finally, the combination of red, green, and blue emissions from Eu, Tb, and ligand-centred fluorescence was simulated and scrutinised. The generation of white illuminants or reference points showed that a proper combination of the three primary colours is needed to obtain correct gamuts in CIELAB, because of the white adaptation intrinsic to this colour space. D65 and E white points were attainable by looking for the combinations of the three primary colours producing the desired chromaticity values. With these newly defined primaries, overlapping gamuts were found for the three sets of ligands, which makes it interesting for metameric colour matching as anti-counterfeiting security features. By matching the luminance (*Y*) of the white points, it was shown that in principle, the same colours can be reproduced by either set, as long as the colour is located within a region enclosing both gamuts.

The limitations of our computational approach were discussed, and practical issues were addressed and highlighted. The main issues are (1) the choice of the excitation UV light, which can raise the black point of the gamut due to residual visible light or non-black emission of the matrix or medium; (2) inner-filter effects and quenching, which can complicate the combination of individual complexes and therefore spectral data; and (3) the quantum yields, extinction coefficients, and concentration, which can limit the perception of the colour stimuli when values are too low.

This work is important for any colorimetric analysis using Ln ions but could also be extended to other emitters. Colorimetric probes are desirable over probes using absolute emissions or even ratiometric data because of the benefit of having visual data that can be quantitatively measured with simple and cheap colorimeters. The limiting factor is of course the accurate calibration of the measuring instrument in order to obtain reliable colour values.

## Supplementary Materials

The following are available online, Spectral_data.xlsx: file containing the emission spectra used as raw data.
